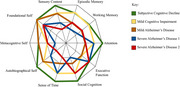# Dimensions of Consciousness in Alzheimer's Disease

**DOI:** 10.1002/alz70857_100648

**Published:** 2025-12-25

**Authors:** Salah Aziz

**Affiliations:** ^1^ Carleton University, Ottawa, ON, Canada

## Abstract

**Background:**

The study of altered conscious states in Alzheimer's disease (AD) often relies on hierarchical models that categorize these states into discrete levels, typically based on unidimensional markers of arousal or awareness complexity. However, these models fail to capture the dynamic, multifaceted nature of consciousness, particularly the global state of consciousness (GSC). A novel construct in consciousness science, GSC refers to an individual's overall conscious condition that modulates the kinds of contents that can enter awareness and the capacity to use them for cognitive and behavioral control. Existing frameworks overlook the complexities of this state. This study critiques these models and proposes a multidimensional framework for GSC in AD.

**Method:**

This work critically examines hierarchical models of consciousness, particularly their limitations in capturing GSC complexities in AD. While grounded in a review of theoretical and empirical studies, the approach moves beyond summarizing existing research. By synthesizing insights into disruptions across cognitive and subjective dimensions—such as sensory content, memory, attention, social cognition, and the sense of self—this work proposes a multidimensional framework for GSC in AD, emphasizing the dynamic interplay between these dimensions.

**Result:**

GSC in AD is a dynamic, non‐uniform state modulating across various phenomenological dimensions. Disruptions in sensory processing, memory, executive function, and the sense of self interact to influence GSC, leading to varying consciousness patterns across individuals. These variations explain fluctuations in awareness and moments of clarity despite disease progression. By integrating these dimensions, the proposed framework offers a nuanced understanding of consciousness in AD, addressing complexities hierarchical models fail to capture.

**Conclusion:**

This study introduces GSC as a novel perspective in AD, emphasizing its dispositional nature and proposing a multidimensional framework to explore the dynamic interplay of cognitive and subjective dimensions. By integrating theoretical and empirical findings, this approach offers a more comprehensive understanding of consciousness in AD, with implications for research and clinical interventions addressing cognitive and existential challenges.